# A Gas Mixture Prediction Model Based on the Dynamic Response of a Metal-Oxide Sensor

**DOI:** 10.3390/mi10090598

**Published:** 2019-09-11

**Authors:** Wei-Chih Wen, Ting-I Chou, Kea-Tiong Tang

**Affiliations:** Department of Electrical Engineering, National Tsing Hua University, Hsinchu 30013, Taiwan

**Keywords:** metal-oxide sensors, dynamic response, temperature modulation, mixture gas prediction

## Abstract

Metal-oxide (MOX) gas sensors are widely used for gas concentration estimation and gas identification due to their low cost, high sensitivity, and stability. However, MOX sensors have low selectivity to different gases, which leads to the problem of classification for mixtures and pure gases. In this study, a square wave was applied as the heater waveform to generate a dynamic response on the sensor. The information of the dynamic response, which includes different characteristics for different gases due to temperature changes, enhanced the selectivity of the MOX sensor. Moreover, a polynomial interaction term mixture model with a dynamic response is proposed to predict the concentration of the binary mixtures and pure gases. The proposed method improved the classification accuracy to 100%. Moreover, the relative error of quantification decreased to 1.4% for pure gases and 13.0% for mixtures.

## 1. Introduction

Estimating the gas concentration is essential to obtain detailed information for analysis. In real scenarios, most target gases are mixtures. Therefore, distinguishing each component in the mixture is important. A standard method of distinguishing components in a gas mixture is gas chromatography-mass spectrometry (GC-MS); however, GC-MS is expensive and time-consuming. Optical methods, despite their cost and complexity, have great performance in terms of sensitivity [1]. The matter of the determination of concentration of single components in a complex mixture is a hot topic [2] and new methods and algorithms for these purposes are in developing [3].

Metal-oxide (MOX) sensors, which are a type of gas sensors, are widely used for gas classification and quantification due to their high sensitivity [4] and low cost. However, different proportions of the mixture cause different responses in MOX sensors. Collecting all the mixtures for training is a costly option. To resolve this issue, a model with a finite dataset can be built to predict unseen data. The performance of predictions depends on the selectivity of the features that comprise the model. MOX sensors also exhibit low selectivity to gases because they usually respond to multiple gases. A multisensor system may increase the selectivity because the cross sensitivity between different sensors compensates for their individual drawbacks [5,6,7]. Gas selectivity can be improved by modulating the temperature of the sensor film to change its chemical reaction rate and obtain additional features [8,9,10,11,12]. By modulating the temperature, the response of the sensor to different gases can reflect the chemical characteristics and reveal additional information for achieving increased selectivity [13]. Therefore, the features with increased selectivity obtained through temperature modulation may contribute to the gas prediction model.

In 2018, researchers reported to use a single sensor to predict the mixture concentration with the peak response under temperature modulation and proposed a linear-quadratic mixture model suitable for the features [14]. However, the peak features were insufficient for classifying the pure gases and mixtures. Consequently, a low classification accuracy was achieved, which decreased the accuracy of the gas quantification. Compared with the peak response under temperature modulation, the dynamic response reflects various chemical reactions at different temperatures and provides more feature diversity for enhancing classification [15,16]. Therefore, this paper proposes using the dynamic response under temperature modulation by heater voltage to accurately quantify and identify methanol, ethanol, and their mixtures. A corresponding feature extraction method and a mixture model for a dynamic response are also proposed to optimize the accuracy of quantification and classification.

## 2. Experiment and Method

### 2.1. Temperature Modulation

In the temperature-modulated method, the heaters of MOX sensors are applied with the voltage waves to heat the sensing film. As the temperature of the sensing film reaches to a certain degree (e.g., 300°C), the reactions between the target gas and the sensing film can be described with a reduction-oxidation reaction [17]. The rate constant, which affects the reaction rate, depends on the temperature. With changes in the temperature, the total concentration of electrons on MOX sensors increases or decreases according to the reaction rate. More electrons result in a higher conductance, and vice versa. Thus, the conductance of the sensors changes.

**Figure 1:**
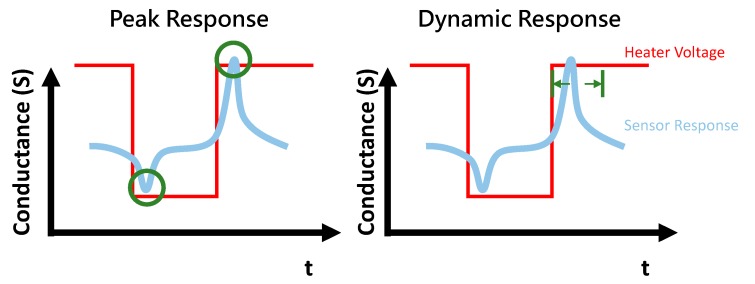
Different methods of extracting information from the temperature modulation. The red line denotes the heater voltage; the blue line denotes the response of the sensor; and the green line denotes the information extracted through different methods.

As an MOX sensor is heated, the conductance of the sensor increases until the temperature reaches the optimum catalytic oxidation temperature and then begins to decrease [17]. As different reducing gases react at different rates, there exist different peaks on the conductance curve. Numerous methods are available for extracting information from the response with temperature changes. As displayed in Figure 1, the peak response represents the maximum or minimum response as the heater voltage switches dramatically. The dynamic response represents the temporal response as the temperature of the sensor changes. The dynamic response contains the peaks, which correlate with the chemical characteristics of the gases. The value of the response depends on the gas concentrations.

### 2.2. Experimental Setup

**Figure 2:**
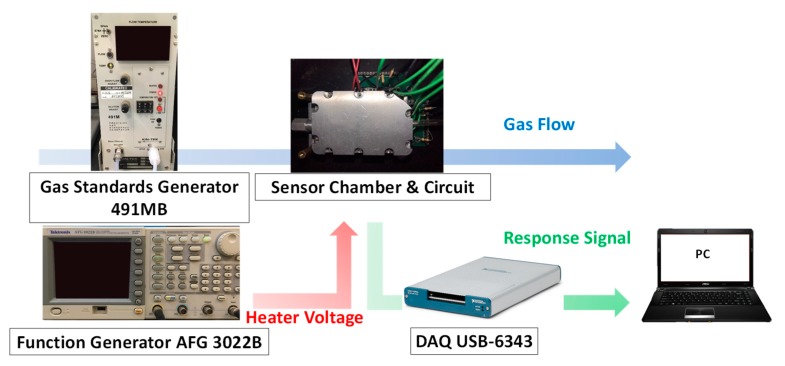
Experimental setup of the sensing system.

This study aimed to classify mixture of two gases: ethanol, and methanol. The gas samples were generated using a standard gas generator (491MB, Kin-Tek, La Marque, TX, USA), and dry air was used as the reference gas. The gases were injected into a metal chamber that contained a MOX sensor (TGS2611, Figaro, Osaka, Japan) with a constant flow rate of 0.2 L/min. The relative humidity was controlled around 70% ± 3%. The signals of the sensor response were acquired using a data acquisition card (DAQ USB-6343, National Instruments, Austin, TX, USA) with a sampling rate of 10 Hz. In the temperature-modulated method, the sensor temperature was related to the heater supply voltage, which was generated and controlled through a function generator (AFG 3022B, Tektronix, Beaverton, OR, USA). Figure 2 depicts the experimental setup of the system.

**Figure 3:**
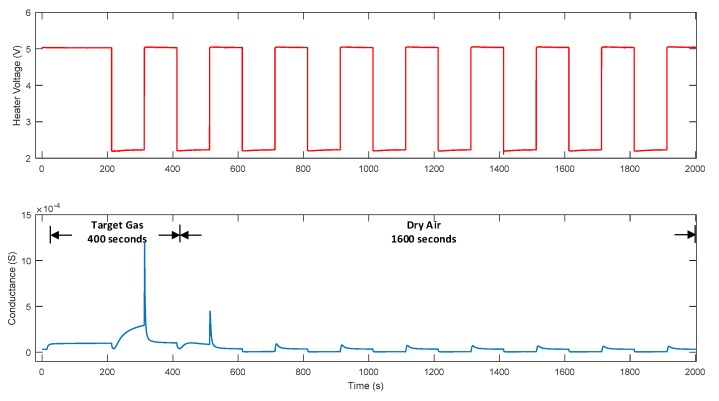
Experiment flow in each measurement.

In this research, a square-wave signal of 2.2–5 V was applied as the heater voltage. The corresponding temperatures of the sensor were 107.3 °C and 307.8 °C, measured by thermocouple. The frequency of the wave was 5 mHz to allow the sensor to reach its steady state after the temperature changed. The response of the sensor reacting with the target gas and dry air under temperature modulation is illustrated in Figure 3. The red line denotes the heater voltage applied to the sensor, and the blue line denotes the response of the sensor. In each measurement, the target gas was injected into the chamber for 400 s, and the heater voltage was applied after the target gas reached the steady state at a constant temperature. The chamber was then purged with dry air for 1600 s to clean the residuals.

The conductance of the gas sensor (*G*) was calculated as follows:

(1) $$G=\frac{1}{R}=\frac{1}{R_{L}\left(\frac{V_{C}}{V_{o}}-1\right)}$$

where $R_{L}$ is the load resistance, $V_{C}$ is the circuit voltage, and $V_{o}$ is the output voltage. Four different concentrations of ethanol gas, four different concentrations of methanol gas, and seven different proportions of mixtures with ethanol and methanol were prepared. The test samples are listed in Table 1.

### 2.3. Feature Extraction

**Figure 4:**
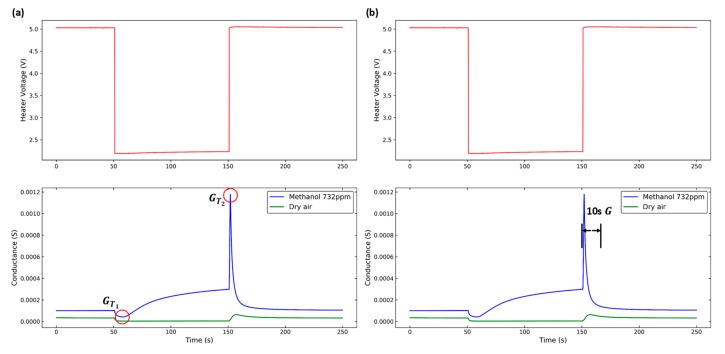
Feature extraction methods: (**a**) Peak response and (**b**) dynamic response.

In this paper, two feature extraction methods are discussed: peak response and dynamic response. The supply voltage waveform of the sensor heater and the conductance waveform of the sensor are illustrated in Figure 4. The red line denotes the heater voltage applied to the sensor; the blue line denotes the response with 732 ppm methanol; and the green line denotes the response with dry air, which was used as a baseline. According to the method in Madrolle’s study, the values of the peak height were acquired as peak responses $G_{T_{1}}$ and $G_{T_{2}}$ when the heater voltage switched down and up [14]. When the sensor was heated, the conductance response in the first 10 seconds was acquired as the dynamic response *G*.

The physical meanings between the two feature extraction methods were different. The ratio of conductance (${\frac{G_{g}}{G_{0}}\;|}_{T_{i}}$) and the difference in conductance ($△G_{i}$) are conventional methods. The ratio of conductance was used to determine the peak response [14]. The difference in conductance could be directly identified from the concentration of electrons on the sensors. The parameter △G is related to the gas concentration through the following power law [17]:

(2) $$△G=G_{g}-G_{0}=aC^{n}$$

where *C* is the concentration of gases, $G_{g}$ is the conductance of the sensor reacting with target gases, $G_{0}$ is the conductance of the sensor reacting with dry air, and *n* is the power coefficient, which depends on the temperature. The aforementioned equation was proved to be highly correlated with the dataset in this study. This characteristic is helpful for identifying and quantifying pure gases.

### 2.4. Dynamic Response

**Figure 5:**
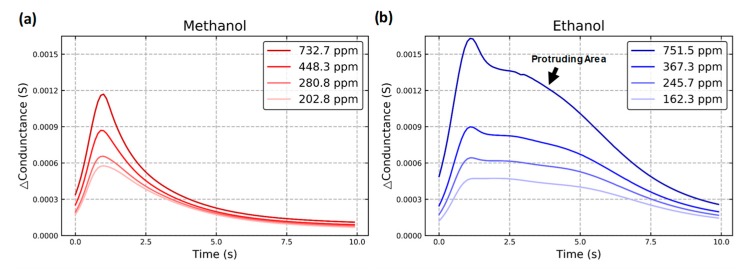
Dynamic response with (**a**) methanol and (**b**) ethanol.

**Figure 6:**
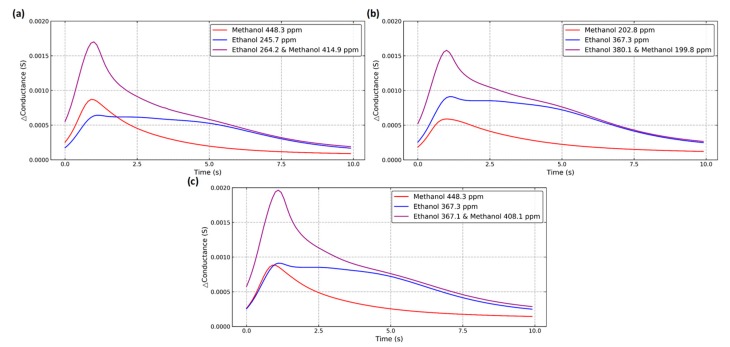
Dynamic response with the mixture: (**a**, **c**) Fixed concentration of methanol and an increasing concentration of ethanol; (**b**, **c**) fixed concentration of ethanol and an increasing concentration of methanol.

The dynamic response with different gas concentrations is depicted in Figure 5. In Figure 5a, a peak occurs at 1.5 s for the dynamic response of methanol. In Figure 5b, after a peak at 1.5 s, additional protruding area occurs for the dynamic response of ethanol. For both methanol and ethanol, an increased gas concentration results in an increased response at the same peak position. Figure 6 indicates that the response of the mixture retains the characteristics of its components. Figure 6a and c show the sensor responses with two ethanol concentrations of 264.2 ppm and 367.1 ppm while maintaining the same methanol concentration. Figure 6b and c show the sensor responses with two methanol concentrations of 199.8 ppm and 408.1 ppm while maintaining the same ethanol concentration. An increase in the gas concentration with different types of gas caused different responses at each time point. The components contained in the mixture can be distinguished and quantified from the characteristics of different relationships at each time point.

### 2.5. Mixture Model

The extracted features and mixture models must be expressed as a formula. In a previous study [14], a linear-quadratic model was proposed to indicate the relationship between the gas concentration and the sensor response.

(3) $$G_{i}\left(C_{1},C_{2}\right)=\frac{G_{i}}{G_{0,i}}-1=a_{1,i}C_{1}^{n_{1,i}}+a_{2,i}C_{1}^{2n_{1,i}}+b_{1,i}C_{2}^{n_{2,i}}+b_{2,i}C_{2}^{2n_{2,i}}+d_{3,i}C_{1}^{n_{1,i}}C_{2}^{n_{2,i}}$$

where *i* denotes the *i*^th^ feature. The aforementioned formula assumes that the characteristics of a gas sensor and the gas concentration exhibit a quadratic relationship. The response with a mixture is the sum of individual responses with different gases and an interaction term. The interaction term represents a possible nonlinear relationship between gases. For a mixture without target gases, the equations are consistent because the ratio of conductance is equal to 1. Since the characteristic of the dynamic response is very different from the peak response, the linear quadratic model may not be enough to model the dynamic response.

To take the dynamic response into account, this study proposes a new formula that assumes that the sensor characteristics and gas concentration of a pure gas exhibit the power law relationship. The value of the response with a mixture is the sum of the individual responses with different gases and an interaction term. In this study, the power law is introduced, and a polynomial interaction term model is proposed. A cubic polynomial as an interaction term indicates the nonlinear relationship among the gases on the sensor. The sensor conductance (△$G_{i}$) has a nonlinear relationship with the methanol concentration $(C_{1}$) and ethanol concentration ($C_{2})$.

(4) $$G_{i}\left(C_{1},C_{2}\right)=△G_{i}=a_{1,i}C_{1}^{n_{1,i}}+b_{1,i}C_{2}^{n_{2,i}}+d_{1,i}C_{1}^{n_{1,i}}C_{2}^{n_{2,i}}+d_{2,i}C_{1}^{2n_{1,i}}C_{2}^{n_{2,i}}+d_{3,i}C_{1}^{n_{1,i}}C_{2}^{2n_{2,i}}$$

Because additional interaction terms are used to represent their nonlinear relationships, additional coefficients can be obtained to distinguish the nonlinear effects caused by methanol and ethanol, which helps to increase the quantization accuracy.

The coefficients $\hat{a},\hat{b},\hat{d},\mathrm{and}\;\hat{n}$ were calculated from the training data. The least-squares method, Levenberg-Marquardt algorithm [14], was used to obtain the optimal local solution.

(5) $$\hat{a},\hat{b},\hat{d},\hat{n}=\underset{a,b,d,n}{\arg\min}\overset{N}{\underset{j=1}{\sum }}{\left(G_{i}\left(C_{1},C_{2}\right)-x_{i,j}\right)}^{2}$$

### 2.6. Model Inversion

From the known reaction value $t_{i}$ in the test data, the gas concentrations $C_{1}$ and $C_{2}$ were calculated using the Levenberg-Marquardt [18] method as the optimization algorithm for obtaining the local optimal solution.

(6) $$\hat{C_{1}},\hat{C_{2}}=\arg\underset{C_{1},C_{2}}{\min}\overset{m}{\underset{i=1}{\sum }}{\left(G_{i}\left(C_{1},C_{2}\right)-t_{i}\right)}^{2}$$

By using the mixture model mentioned in Section 2.5, the known gas concentration was used to restore the *i*^th^ feature points in training stage. If the mixture model was fitted on each feature, the gas concentration could be predicted using the known sensor response in testing stage. The coefficients calculated with the features affected the performance for classifying and quantifying the gas. Features with increased selectivity help allow superior classification and quantification results to be obtained.

### 2.7. Performance Parameter

The coefficient of determination $\left(R^{2}\right)$ score was used to determine the correlation between the equation and the actual measurement. The value of the $R^{2}$ score is between 0 and 1. The closer the value is to 1, the higher is the correlation.

(7) $$R^{2}\;\mathrm{score}=1-\overset{N}{\underset{i=1}{\sum }}\frac{{\left(x_{\mathrm{predict},i}-x_{\mathrm{true},i}\right)}^{2}}{{\left(x_{\mathrm{true},i}-\bar{x_{i}}\right)}^{2}}$$

where $x_{\mathrm{predict}}$ denotes the predicted feature,$\;x_{\mathrm{true}}$ denotes the true feature, $\bar{x}$ denotes the mean value of x, and *N* denotes the number of samples. The relative error (in %) was used to examine the concentration estimation.

(8) $${\mathrm{Mean}\;\mathrm{relative}\;\mathrm{error}}_{{\mathrm{Class}}_{j}}\;\left(\%\right)=\frac{1}{N_{{\mathrm{Class}}_{j}\;}}\underset{i\in {\mathrm{Class}}_{j}}{\sum }\frac{\left|y_{\mathrm{predict},i}-y_{\mathrm{true},i}\right|}{y_{\mathrm{true},i}}$$

where $y_{\mathrm{predict}}$ denotes the predicted concentration of the gas,${\;y}_{\mathrm{true}}$ denotes the true concentration of the gas, ${\mathrm{Class}}_{j}$ denotes the $j^{\mathrm{th}}$ class of the gas (methanol, ethanol, or their mixtures), and *N* denoted the total amount of the class. The relative error was suitably representative of the accuracy of the algorithm because the samples in this study were all high-concentration gases. In the error calculation performed in this study, the classification results of the prediction data were assumed to be correct so that the zero error of calculating the relative error of pure gases could be avoided. There existed four categories of gases: methanol, ethanol, methanol in the mixture, and ethanol in the mixture. The pure gas and mixture were observed separately, and the differences among the mixture and the pure gases were clearly indicated by the error.

## 3. Results

### 3.1. Data Distribution

**Figure 7:**
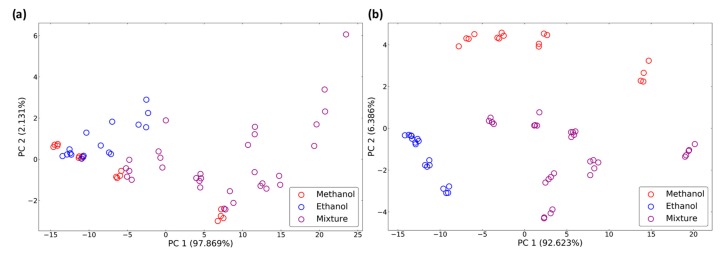
Principle component analysis (PCA) results for data distribution of (**a**) peak features and (**b**) dynamic features.

The peak features and dynamic features were compared on a two-dimensional map. The data distribution can be used to determine the classification ability of each feature. Figure 7a shows principle component analysis (PCA) [19] results of the data distribution using the peak features. The data points in this figure are mixed together due to a lack of selectivity for methanol and ethanol. Figure 7b shows PCA results of the data distribution using the 100-dimension dynamic features. The different categories of gases were linear-separable, and the data distribution may significantly increase the classification accuracy.

### 3.2. Mixture Model

**Figure 8:**
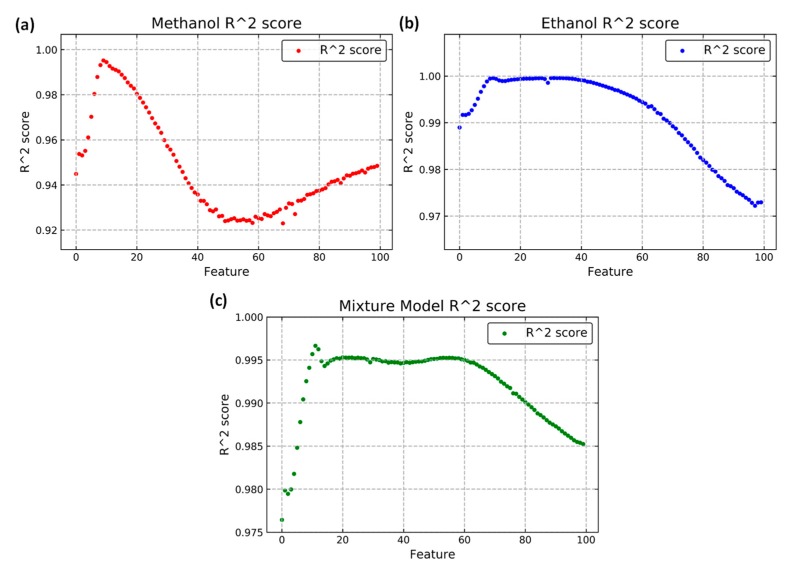
*R*^2^ score obtained with the power law for each gas: (**a**) Methanol and (**b**) ethanol; (**c**) *R*^2^ score obtained with the mixture model for all data.

The power law is proposed to relate the concentration of pure gases with the dynamic features. The $R^{2}$ score was used to determine the correlation between the equation and the features. In Figure 8, all the features are highly correlated with Equation (2) due to the high $R^{2}$ score. Thus, the concentration of the pure gases could be predicted accurately with the dynamic features. The $R^{2}$ score was calculated with all the data for the proposed model in Figure 8. The added interaction terms and the high-correlation relationship may cause the overfitting problem. Therefore, the leave-one-out method was implemented to verify the model.

### 3.3. Results of Predictions

In this section, the accuracies of the concentration predictions for methanol, ethanol, and their mixtures were compared using different characteristics and different mixture models. The leave-one-out method was used for the predictions, and the concentrations of the pure gases were verified through interpolation. Two methanol concentrations, two ethanol concentrations, and seven different proportions of mixtures were selected to verify the model. Thus, verification was performed 11 times. Table 2 and Table 3 present the prediction results with different variables. The error is expressed by the mean relative error. In Table 2, the error rate in the bold green font is the result obtained using the method in Madrolle’s study [14]. Table 2 and Table 3 indicate that the use of the dynamic response can significantly improve the quantization accuracy compared with the use of the peak response. In Table 3, the error rate in the bold red font is the result obtained using the method proposed in this paper. The accuracy of the prediction with the dynamic response could be optimized using the difference of conductance and the polynomial interaction term model.

**Figure 9:**
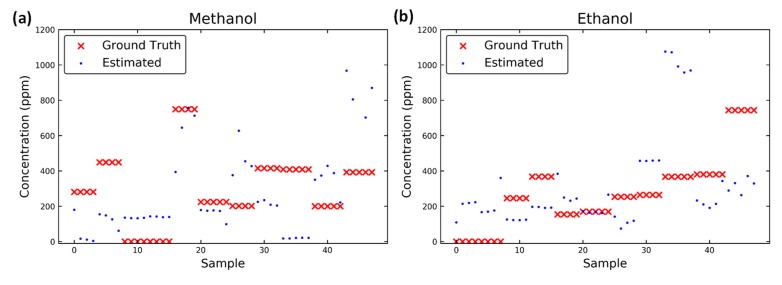
Estimated concentration of (**a**) methanol and (**b**) ethanol in each sample obtained with the method in Madrolle’s study [14]. The ground truth in red is presented for a comparison with the estimated results.

The ground truth and the estimated data obtained for each test when using the linear-quadratic model with the peak response, which is characterized by the ratio of conductance values are illustrated in Figure 9. When the peak features were used, pure gases were misclassified as mixtures and mixtures were misclassified as pure gases. Although the misclassification of pure gases as mixtures did not affect the accuracy of quantification, it may affect subsequent judgments. However, the misclassification of mixtures as pure gases may have a considerable influence on the accuracy of mixture quantification.

**Figure 10:**
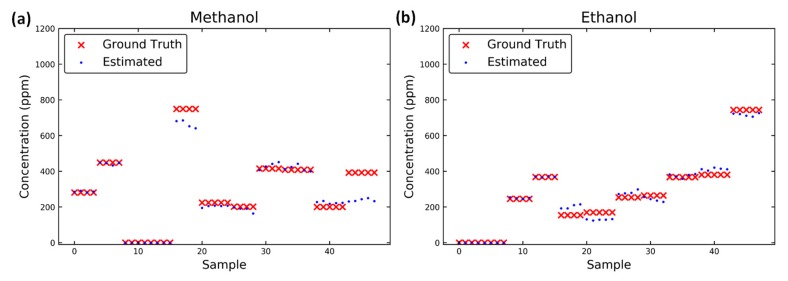
Estimated results for (**a**) methanol and (**b**) ethanol in each sample obtained with the proposed method. The ground truth in red is presented for a comparison with the estimated results.

The ground truth and the estimated data of each test when using the polynomial interaction term model with the dynamic response, which is characterized by the difference in conductance values and are depicted in Figure 10. When using the dynamic features, the problem of misclassification did not occur, which proves that the selectivity is considerably improved by the dynamic response. Moreover, the consistency of the prediction results increased with additional features in the dynamic response, which may decrease the effect of noise.

The accuracy of the MOX sensors is defined as the difference between the results and the true value. The precision of the MOX sensors is defined as the difference between each result. The mean relative error in the prediction model was depicted in Equation (8). The accuracy and precision of the MOX sensors both affect the component $y_{\mathrm{predict},i}$ in the equation. The higher accuracy makes $y_{\mathrm{predict},i}$ closer to $y_{\mathrm{true},i}$, thus reduces the mean relative error. The higher precision makes $y_{\mathrm{predict},i}$ clustering together, thus also reduces the mean relative error.

## 4. Conclusions

In this study, different concentrations of ethanol, methanol, and various ratios of their mixtures were identified and quantified according to sensor dynamic response by temperature modulation. The three categories in the data distribution were linear-separable with the principle component of the dynamic response. To increase the quantification accuracy with the dynamic response, the highly correlated relationship of the power law was verified. This paper proposes the use of a cubic polynomial model to fit the features of the mixture gas. The leave-one-out method was used to verify the proposed technique, and the success rate for classification was 100%. The problem of misclassification in previous research was solved. The prediction error of the gas was controlled to within 1.4%, and the prediction error for the mixture was controlled to within 13.0%. In the future, additional sensors can be added into an array to increase the selectivity for an increased number of gases in various applications. Moreover, feature selection methods can be implemented to reduce the amount of features that contain duplicated or redundant information.

## Figures and Tables

**Table 1:** List of test samples.

Gas Type	Methanol Concen-Tration (ppm)	Ethanol Concen-Tration (ppm)	Amount	Gas Type	Methanol Concen-Tration (ppm)	Ethanol Concen-Tration (ppm)	Amount
Ethanol	0	162.3	5	Mixture	392.6	743.8	5
Ethanol	0	245.7	4	Mixture	199.8	380.1	5
Ethanol	0	369.3	4	Mixture	408.1	367.1	5
Ethanol	0	751.5	4	Mixture	414.9	264.2	4
Methanol	202.8	0	4	Mixture	201.4	253.4	5
Methanol	280.8	0	4	Mixture	224.3	169.1	4
Methanol	448.3	0	4	Mixture	748.8	154.0	4
Methanol	732.7	0	4				

**Table 2:** Prediction results of the peak response with different variables.

Class	Peak Response (2 Points)			
	Linear-Quadratic Model		Polynomial Interaction Term Model	
	$\frac{G}{G_{0}}-1$	$G-G_{0}$	$\frac{G}{G_{0}}-1$	$G-G_{0}$
	Mean Relative Error (%)			
Methanol	77.0	71.2	18.0	12.7
Ethanol	48.6	31.3	71.5	76.8
Methanol in mixture	76.7	48.4	88.0	92.1
Ethanol in mixture	70.6	51.1	72.3	118.1

**Table 3:** Prediction results of the dynamic response with different variables.

Class	Dynamic Response (100 points)			
	Linear-Quadratic Model		Polynomial Interaction Term Model	
	$\frac{G}{G_{0}}-1$	$G-G_{0}$	$\frac{G}{G_{0}}-1$	$G-G_{0}$
	Mean Relative Error (%)			
Methanol	32.9	13.4	18.7	1.4
Ethanol	29.2	6.7	35.0	1.3
Methanol in mixture	32.7	20.3	30.0	13.0
Ethanol in mixture	15.3	14.2	29.0	12.5
